# CRISPR/Cas9 System: A Bacterial Tailor for Genomic Engineering

**DOI:** 10.1155/2018/3797214

**Published:** 2018-09-18

**Authors:** Bilal Ahmad Lone, Shibendra Kumar Lal Karna, Faiz Ahmad, Nerina Shahi, Yuba Raj Pokharel

**Affiliations:** Faculty of Life science and Biotechnology, South Asian University, Akbar Bhawan Chanakyapuri, New Delhi 110021, India

## Abstract

Microbes use diverse defence strategies that allow them to withstand exposure to a variety of genome invaders such as bacteriophages and plasmids. One such defence strategy is the use of RNA guided endonuclease called CRISPR-associated (Cas) 9 protein. The Cas9 protein, derived from type II CRISPR/Cas system, has been adapted as a versatile tool for genome targeting and engineering due to its simplicity and high efficiency over the earlier tools such as ZFNs and TALENs. With recent advancements, CRISPR/Cas9 technology has emerged as a revolutionary tool for modulating the genome in living cells and inspires innovative translational applications in different fields. In this paper we review the developments and its potential uses in the CRISPR/Cas9 technology as well as recent advancements in genome engineering using CRISPR/Cas9.

## 1. Biology of CRISPR/Cas9 System

**Figure 1:**
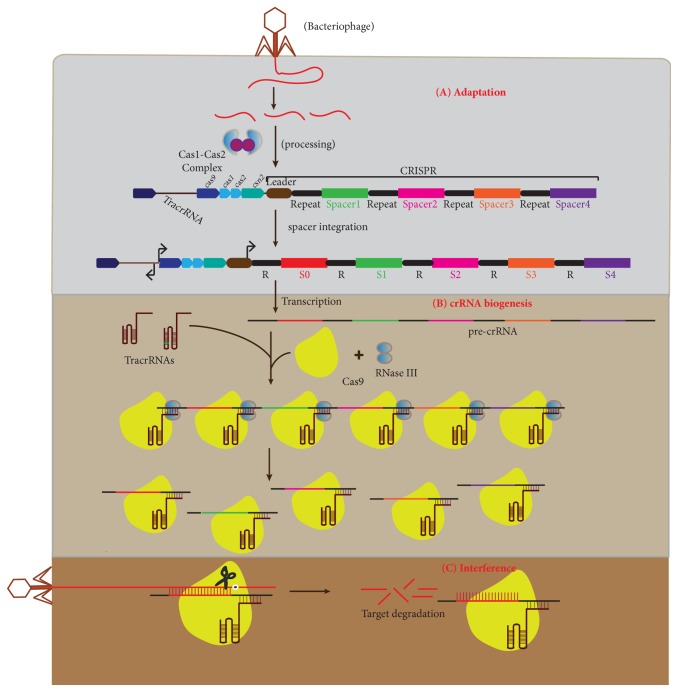
**An overview of type II CRISPR/Cas immunity. **The CRISPR/Cas system provides the adaptive immunity to prokaryotes against the foreign DNA in three phases.** (A)** Adaptation: during the adaptation phase, the Cas1-Cas2 complex selects the new spacer (red) and integrates it into the leader-proximal end of CRISPR locus.** (B) **crRNA biogenesis: in this phase, the CRISPR locus is transcribed into pre-crRNA that forms duplexes with tracr-RNAs with repeat-anti-repeat interaction followed by recognition and cleavage by RNase III into mature crRNA in the presence of Cas9.** (C) **Interference: during this phase, the mature crRNA/tracr RNA hybrid that remains bound to Cas9 acts as a guide for Cas9 to recognize and degrade the foreign DNA upon subsequent infection.

The CRISPR/Cas9 system is a prokaryotic nucleic acid-based adaptive immune system that enables selected microbes to respond to and eliminate foreign genetic material [1]. Microbes that have been exposed to foreign genetic material through transduction, conjugation, and transformation are stimulated to establish defence mechanisms that identify foreign DNA and protect themselves against genome invaders [1, 2]. Defence is acquired by integrating short fragments of foreign DNA into CRISPR region [3] (Figure 1(A)). The CRISPR region contains short repetitive base sequences separated by stretches of variable sequences referred to as spacers that share the sequence homology with foreign elements including bacteriophage and plasmid [4]. Alteration of CRISPR locus by addition and deletion of spacers determines the resistance and sensitivity to phages, respectively. Cas genes, which encode Cas proteins, usually flank CRISPR array that is preceded by AT-rich leader sequence [4, 5].

CRISPR immunity in microbes is acquired through (A) adaptation or spacer acquisition, (B) CRISPR-RNA (crRNA) biogenesis, and (C) target interference [5, 6] (Figure 1). During adaptation phase, invading DNA is spliced into small fragments and incorporated into a CRISPR locus as new spacers that become the memory record of infection. Integration of new spacers in response to DNA infection is polarized towards the leader end of the CRISPR locus [1, 7]. The analysis of the protospacer (sequence within the invading nucleic acid that shares the sequence homology with spacer sequence) revealed the presence of a short 2-3 nucleotide conserved sequence adjacent to protospacer, referred to as “CRISPR motif” or “the protospacer adjacent motif”(PAM) [4, 8, 9]. The PAM sequence is essential for selection and acquisition of protospaces into the CRISPR array by Cas1 and Cas2 protein complexes [10, 11] (Figure 1(A)).

In the crRNA biogenesis phase, CRISPR array is transcribed into precursor CRISPR-RNA (pre-crRNA) followed by maturation to crRNAs, each containing a specific spacer sequence flanked by short RNA sequences [12]. Presence of tracr RNA, RNase III, and Csn1(Cas9) is important for the processing of pre-CrRNA into mature cr-RNA. The mature crRNA-tracrRNA hybrid remains firmly associated with Cas9 to form a complex for target interference [13–15].

During the interference phase, the crRNA in the Cas9-crRNA-tracrRNA ribonucleoprotein (crRNP) complex base pairs with the corresponding protospacer and stimulates Cas9 for the recognition and destruction of the matching sequence by cleaving both strands of the target [16]. Cas9 protein cleaves the protospacer at a site that is located 3 bases upstream of the protospacer adjacent motif [17]. The absolute requirement of the PAM sequence for the cleavage of the protospacer excludes the “autoimmune” response within the CRISPR locus as the host locus lacks the PAM sequence.

## 2. Exploitation of CRISPR/Cas9 System for Genomic Engineering

**Figure 2:**
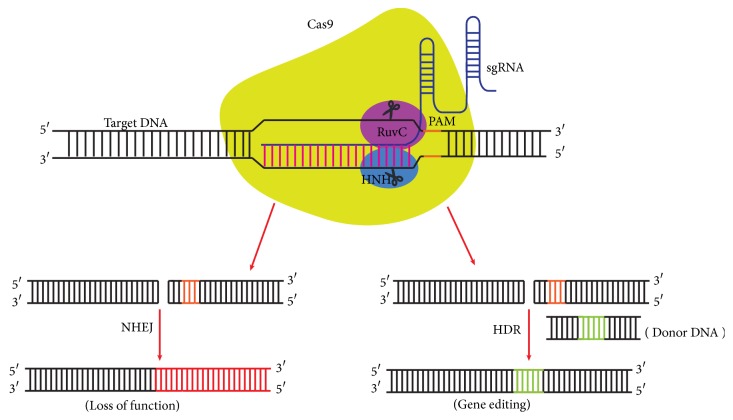
**An overview of CRISPR/Cas9 mediated genome editing.** The sgRNA that comprises a single strand RNA guides the Cas9 protein to the target DNA site with a sequence complementary to the 5′ end of sgRNA. The PAM dependent recognition of target DNA sequence by Cas9 initiates the DNA cleavage at a specific site 3 bp upstream of the PAM. The double-strand break generated by Cas9 can be repaired by either NHEJ or HDR. The NHEJ repair often results in indel mutation and inactivation of the gene while the HDR allows the high-fidelity precise genome editing when supplied with donor template.

The CRISPR/Cas9 system is acquired from prokaryotes and exploited for efficient and reliable technology that makes precise and targeted editing of the genome of living cells [39]. The Cas9 nuclease in complex with crRNA-tracrRNA duplex induces the double-strand break within the target DNA that is a prerequisite for genome editing [16, 40] (Figure 2). The CRISPR/Cas9 system can achieve site-specific DNA recognition and cleavage in mammalian cells through the reconstitution of the heterologously expressed human codon-optimized Cas9 and the key RNA components (crRNA and tracrRNA) [40]. The modified version of the CRISPR/Cas9 system in mammalian cells comprises the codon-optimized Cas9 protein with nuclear localization sequence (NLS) that drives the nuclear compartmentalization in mammalian cells [39, 40]. NLSs flanking Cas9 are more efficient in targeting Cas9 to the nucleus [40]. Furthermore, crRNA and tracrRNA are fused together to create the chimeric single guide RNA (sgRNA) via an engineered loop (tetraloop) that retains the features of crRNA-tracrRNA duplexes for DNA target recognition and Cas9 recruitment [16, 39] and also retains the efficient capability of inducing the Cas9 mediated double-strand break [41]. For the gene knockout in a mammalian system, the templates for the crRNA and tracrRNA or the combined sgRNA are cloned into the Cas9 expression plasmids or into the separate plasmids driven by either U6 or H1 promoters for transcription [39, 40]. The crRNA sequence of sgRNA contains the guide (20 nt) and repeat regions (12 nt), whereas the tracrRNA sequence is divided into anti-repeat (14 nt) and three stem loops [42]. The sgRNA with tracr +67 or +85 nucleotide is more efficient and versatile for cleaving the target sites than corresponding crRNA-tracrRNA duplex. Cas9 activity is also affected by the position of mismatches within the guide sequence as mismatches with the PAM-proximal 8-12 bp of the guide sequence are less tolerant than the PAM-distal counterpart [43].

**Figure 3:**
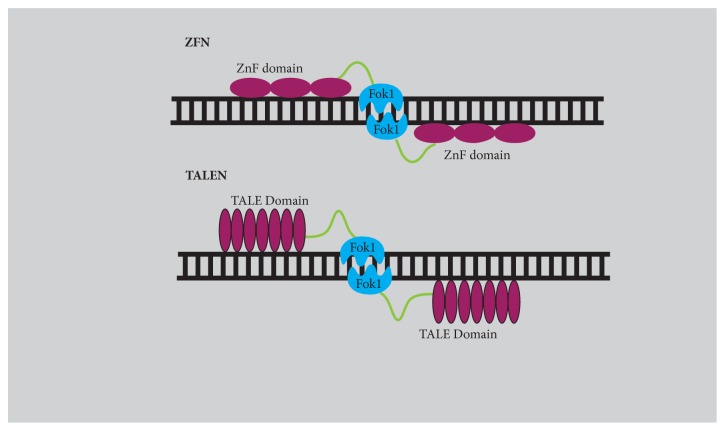
Zinc Finger Nuclease (ZFN). ZFN (discovered by Chandrasegaran and his team in 1996) are sequence-specific chimeric proteins containing DNA binding domain fused to nonspecific cleavage domain (derived from type II restriction enzyme* FOKI*) [18–20]. DNA binding domain consists of 3-6 Cys_2_-His_2_ tandemly arranged zinc finger repeats that recognize 9-18 bp sequences (3 bp by each ZFN unit) [21]. Each finger contains approximately 30 amino acids with one *α* helix and two *β* strands [19]. The chimeric ZFN are engineered to assemble in pairs and enable efficient and precise genetic modifications by inducing DSBs. In addition to dimerization of FokI nuclease domains, ZFN requires correct spacer sequence (5-6 bp) and orientation of chimeric nucleases for the cleavage of dsDNA [22, 23]. Despite wide applications, the major challenge was to increase the specificity since it was cleaving off-targets that had sequence homology to on-target making ZNF cytotoxic [24]. Custom designed ZNF are prepared by altering DNA binding domain and catalytic domain through mutagenesis and modular assembly of precharacterized ZNF [25]. Till date it has been used for genome editing in mice [26], insects [27], zebrafish [28], and humans (embryonic cells and induced pluripotent stem cells) [29]. Transcription activator-like effector Nuclease (TALEN). TALENS derived from the plant pathogen* Xanthomonas *sp. are virulence proteins which consist of DNA binding domain and FOK1 nuclease domain. These domains act as dimers and bind to the opposite strands of DNA, separated by a spacer sequence, and create a double-stranded break. DNA binding domain consists of 33-35 amino acid repeats and is arranged in tandem [30, 31]. These repeats are similar except for two highly variable amino acids at positions 12 and 13 called repeat variable di-residue (RVD) which are responsible for specific base recognition and engineering of these bases in repeats [32]. A total four of RVD modules can recognize each of the bases guanine (G), adenine (A), cytosine (C), and thymine (T) and each module is able to function independently. The target sequence must contain thymine (T) at the 5′ end for recognition by TALENS and the spacer sequence should be of 12-20 bp between the dimers [31]. Compared to ZNF, TALENS possess reduced cytotoxicity, are high on targeting efficiency, and are easy to design. However, its high molecular weight makes it difficult for the delivery in the nucleus. AAV vectors are generally used for the delivery of TALENs due to their low immunogenicity and less oncogenic risk [33]. TALENS have been applied for gene disruption in Drosophila [34],* C. elegans *[35], Arabidopsis [36], Zebrafish [37], and human embryonic stem cells [38].

Site-specific cleavage of target DNA by CRISPR/Cas9 system provides new ways to engineer genomic DNA [54] (Figure 2) both* in vivo *[55] and* in vitro* [39, 56]. Genomic editing by CRISPR/Cas9 system offers considerable advantages over earlier genome editing tools, such as ZFN and TALENs that share the same principle of programming the nuclease to a specific sequence within the genome to induce a double-strand break (Figure 3). In contrast to ZFN and TALEN that require substantial protein engineering, the CRISPR/Cas9 system needs only replacing the 20-nucleotide length guide sequence to bind it to a new target site. Moreover, the CRISPR/Cas9 system can facilitate multiplex genome editing by simply introducing a combination of sgRNAs [39]. CRISPR/Cas9 represents a system that is easier to design, inexpensive, efficient, and capable of high performance. Induction of sequence-specific DSB stimulates the DNA repair mechanisms to repair the DSB by one of at least two different pathways: nonhomologous end joining (NHEJ) and homology-directed repair (HDR) [57, 58] that can be exploited for targeted genome editing. DSB repaired by NHEJ can create disruptive insertions and deletions at target sites, cause a shift in the reading frame of the coding region, and result in gene knockout. The HDR of DSB with donor template facilitates complex genome engineering, which allows the targeted insertion or replacement of the DNA sequence by recombination. However the donor template must contain homologous sequences flanking the genetic segment to be incorporated into the target region. The CRISPR/Cas9 system in combination with two sgRNAs robustly increases the efficiency of gene editing via homologous recombination in the presence of a donor template compared to single sgRNA [39].

## 3. Mechanism of Specific DNA Cleavage by CRISPR/Cas9

**Figure 4:**
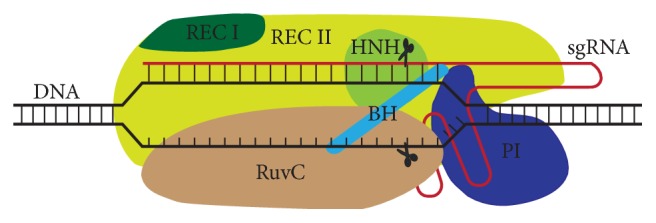
**Cas9-sgRNA-DNA complex. **Cas9 protein is an architecture of multiple domains: RecI, RecII, Bridge helix, PAM-interacting, RuvC, and HNH, which accommodates negatively charged sgRNA-DNA heteroduplex. The complex triggers the cleavage of target DNA when sufficient RNA-DNA complementarity is available for the activation of HNH and RuvC nuclease domains.

Cas9, a programmable RNA guided endonuclease, is the most widely used endonuclease for genomic editing among the several Cas proteins available. The Cas9 nuclease has two conserved nuclease domains: HNH and RuvC domains; both generate DSB in target DNA [16]. The crystal of Cas9 shows that it is a bi-lobed structure in which the central nucleic acid recognition (REC) lobe (that consists of bridge helix, Rec1, and Rec2 domains) along with NUC lobe (which consists of RuvC, the HNH, and the PAM-interacting domains) forms a channel to accommodate the negatively charged sgRNA-target DNA heteroduplex [42] (Figure 4).

Cas9 forms a binary complex with the sgRNA by recognizing the PAM-proximal guide region, followed by the loading of the Cas9-sgRNA complex on a target DNA sequence that depends on the presence of a compatible PAM element [59]. The PAM dependent recruitment of the Cas9-sgRNA complex triggers the initiation of the DNA-sgRNA heteroduplex R-loop formation [60]. During the R-loop extension, sgRNA guide sequence complementarity with the target site of DNA strand leads to allosteric activation of HNH and Ruvc nuclease domains that results in the cleavage of the target dsDNA [61]. Recently, it has been reported that the Cas9 REC lobe (REC3) undergoes a conformational change upon interacting with RNA-DNA heteroduplex and brings the transition into the REC2 domain. This RNA-DNA mediated transition in the REC lobe is essential for signalling to the HNH nuclease to regulate overall catalytic competence of Cas9 [62].

The PAM dependent sgRNA-Cas9 mediated destabilization of the target DNA proceeds in a directional manner and allows the adjacent DNA to be searched for complementarity to the 10 to 12 nt “seed” sequence at the 3′ end of guide RNA segment [63]. The presence of the seed sequence determines the target specificity [43] and mismatches in the seed sequence abrogate the cas9 nuclease activity [63]. The PAM sequence readout by Arg1333 and Arg1335 of Cas9 facilitates the interaction of +1 phosphate group with the phosphate lock loop that promotes the melting of the local DNA, and gRNA-target DNA heteroduplex formation in a directional manner [64]. In the absence of the crRNA:tracrRNA duplex, Cas9 enzyme maintains an autoinhibitory conformation. The Cas9-RNA complex searches the target DNA with a random collision that reduces the amount of time spent at off-target. Binding the Cas9-RNA to the PAMs for longer time interrogates the flanking DNA for guide RNA complementary and leads to the activation of Cas9 nuclease domains when it binds to the correct target site [64].

## 4. Strategies for Improving CRISPR/Cas9 Specificity

**Figure 5:**
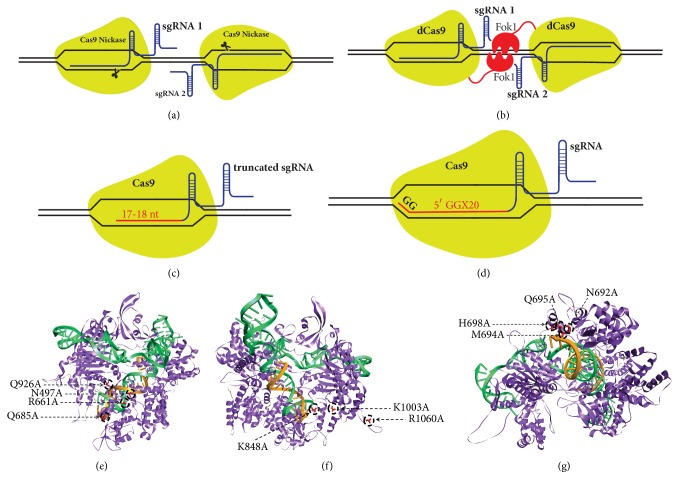
**Strategies for improving the CRISPR/Cas9 specificity. (a)** A pair of sgRNAs guiding Cas9 nickases to bind and nick the opposite DNA strands complementary to gRNA sequences.** (b)** Fusion of catalytically “dead” Cas9 with dimerization-dependent FokI nuclease domains.** (c)** Altering the gRNA to truncated gRNA (truRNA) with only17-18 nucleotides.** (d)** gRNA with two additional guanine nucleotides at the 5′- end to form 5′-GGX20 sgRNA.** Engineered variants of Cas9: (e)** SPCas9-HF1,** (f)** eSpCas9, and** (g)** HypaCas9 (*in silico* mutants were generated from RCSB PDB-4008 using Discovery Studio).

Specificity is the major concern in the CRISPR/Cas9 system as Cas9 can cleave off-target sites that are not fully complementary to the guide sequence of sgRNA [40, 43]. Software tools have been developed for predicting optimized gRNAs with high specificity and relative off-target frequencies (Table 1). In addition to optimized gRNA design, strategies can be employed for minimizing off-target effects: (a) by titrating the amount of Cas9 and sgRNA-DNA delivered. Though, increasing specificity by titrating Cas9-sgRNA concentration also reduces on-target frequencies [43]; (b) wild-type cas9 is mutated to D10 mutant nickase and paired with two sgRNAs to induce nick in the opposite strands of two nearby target sites. This double-nicking strategy reduces the off-target activity by 50- to 1500-fold relative to wild-type without sacrificing the on-target cleavage efficiency [65, 66] (Figure 5(a)); (c) the engineering of dimerization-dependent nonspecific FokI cleavage domains (by fusing FokI monomer with the catalytically inactive dCas9) improves the cleavage specificity fourfold greater compared to paired nickase, when two dCas9-FokI complexes are recruited at similar loci [67, 68] (Figure 5(b)). DNA cleavage by FokI-dcas9 requires the association of two Fok1-dcas9 monomers that simultaneously bind target sites with correct orientation and spacing of 15 or 25 bp (~1.5 or 2.5 helical turns) between the sgRNA pair [67, 69]. The efficient cleavage specificity of FokI-dCas9 is due to the inactivity of the monomeric FokI-dCas9:sgRNA complexes and the correct spacing as well as the orientation of the assembly of a Fok1-dCas9 dimer complex [68]. (d) Cas9 cleavage specificity can be enhanced by using truncated guide RNA with complementarity lengths of 17 to 18 nucleotides (Figure 5(c)). This strategy decreases the undesired mutagenesis by full-length gRNA directed Cas9 at some off-target sites by 5,000-fold or more without affecting the on-target genome editing efficiencies [70]. The combination of “tru-gRNAs” with nickases further reduces the off-target effects [70]. (e) The use of guide RNA with two additional guanine bases at the 5′ end improves more than hundredfold specificities compared to the conventional gRNAs [66]. 5′-GGX20 sgRNA efficiently discriminates on-target sites from off-target sites that differ by ≥2 nt [66] (Figure 5(d)). However, in some cases, the 5′-GGX20 gRNAs can reduce the on-target activity of Cas9 relative to matched standard-length gRNAs; (f) engineering variants of Cas9, Sp-Cas9 HF1[71] and eSpcas9[72], have proven effective in improving genome-wide specificity. Introduction of alanine substitution at four residues (N497A, R661A, Q695A, Q926A) in SpCas9 creates SpCas9-HF1 (high-fidelity variant 1) that disrupts the nonspecific contact between SpCas9 and the phosphate backbone of target DNA sites [71](Figure 5(e)). Substitutions in the SpCas9-HF-1 reduce the “excessive energy” of SpCas9-sgRNA complex while retaining the energy needed for optimal recognition of its intended DNA sites. The SpCas9-HF1 possesses 70% or more activity of wild-type SpCas-9 and reduces all or nearly all genome-wide off-targets as detected by GUIDE-seq analysis. The SpCas9 variant eSpCas9 1.1 (enhanced SpCas9 version 1.1) created by alanine substitution at three positions (K848A/K1003A/R1060A) neutralizes the positively charged residues within the nontarget strand groove (positioned between the HNH, RUV, and PAM-interacting domains) of Cas9 (Figure 5(f)) [72]. Testing of eSpCas9 1.1 for the genome-wide detection of double-strand breaks using BLESS has proven that it reduces all or most off-targets. Recently, Doudna and her group have reengineered the REC3 domain of Cas9 by targeted mutagenesis (N692A/M694A/Q695A/H698A) to produce a hyper-accurate Cas9 (HypaCas9) variant that displays improved target specificity [62](Figure 5(g)). Despite these advancements, still some mutations at off-target sites have been observed and suggest that further optimization of Cas9 is required; (g) an alternative approach for improving the specificity of Cas9 is the direct addition of Cas9 and sgRNA as ribonucleoprotein (RNP) complexes to the cell for CRISPR/Cas9 genome editing [73]. Codelivery of Cas9 and sgRNA allows fast action and efficient genome editing [74] of the RNP complex in the nucleus and enhances the specificity by the rapid clearance of Cas9 RNP complex available for off-target cleavage [73] (Table 2). The off-target effect increases by increasing the nucleic acid-based delivery of Cas9 [43, 75], while delivery of short-lived Cas9 RNP [73] in synchronized cells enhances high-fidelity on-target editing [76]. (h) In another alternate strategy, chemically modified sgRNA bridged nucleic acids at specific locations in crRNA can enhance the specificity of Cas9 several magnitude and reduce the off-targets [77].

Strategies that are used to enhance specificity can be combined to further reduce or abolish the off-target effects. Thus, delivery of Sp-Cas9 HF1/espCas9/ HypaCas9 with 5′-GGX20 /truncated gRNAs or RGNickase RNP with two 5′-GGX20/ truncated gRNAs could make RNA guided nucleases (RGNs) more specific and more efficient.

## 5. Methods for Detection of CRISPR/Cas9 Mediated On-Target and Off-Target Mutations

**Figure 6:**
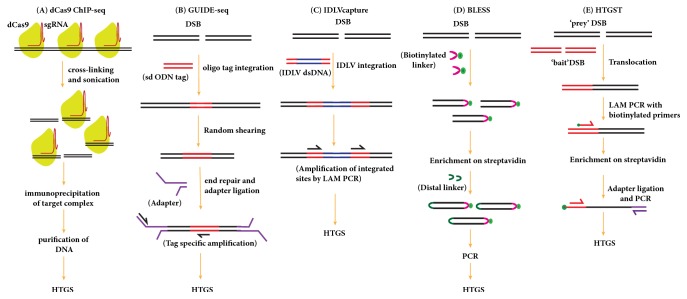
**Genome-wide methods for the detection of off-target sites caused by Cas9 nuclease. (A) dCas9-Chip-seq:** in dCas9-Chip-seq, the sgRNA and catalytically dead Cas9 (dCas9) plasmid are transfected into the cells. dCas9 proteins bound to DNA are immunoprecipitated after the cross-linking and shearing. The immunoprecipitated dCas9-associated DNA is analyzed by HTGS.** (B) GUIDE-seq: **in GUIDE-seq, the DSBs generated by RGN in living cells are tagged by integration of a blunt, short, double-stranded oligodeoxynucleotide (dsODN) followed by unbiased tag amplification and high-throughput sequencing for mapping the off-target cleavage sites. Integration sites are identified by LAM-PCR and high-throughput sequencing.** (C) IDLV capture:** after the transfection of Cas9-sgRNA complexes, the IDLV particles are delivered to get integrated into the RGN induced DSBs. Integration sites are identified by LAM-PCR and high-throughput sequencing.** (D) BLESS:** in BLESS, the RGN induced DSBs are ligated with sequencing adapters followed by fragment enrichment and amplification for high-throughput sequencing.** (E) HTGST**: in HTGST, the RGN generated unknown “prey” sequences are captured by known “bait” sequences by end joining repair of DSBs. The captured bait sequences are subjected to LAM-PCR followed by high-throughput sequencing.

Programmable nuclease can cut their target sites efficiently inducing site-specific DSB in the genome. However, these nucleases can also generate DSBs mutations at off-target sites that differ several nucleotides from on-target sites [45, 66, 78]. Therefore, it is critically essential to validate the on-target and off-target modifications of genomes if the programmable nucleases are used for research and therapeutic applications. On-target mutations (indel or HDR) can be validated by Sanger sequencing, mismatch cleavage assays (CEL-I nuclease and T7 endonuclease I (T7EI) [79, 80], Restriction Fragment Length Polymorphism (RFLP) analysis, or sequencing-based methods [81]. RNA Guided Nuclease (RGN) can cleave at genomic off-target sites with 5′-NGG-3′, 5′-NAG-3′ or 5′-NGA-3′ PAMs [45] that may have detrimental effects on gene expression and can possibly lead to aberrant cellular function. Computational programs are used to predict and rank potential off-target of gRNA. Although many computational programs have been developed to identify off-target sites (Table 1), none of them can predict off-target sites with high accuracy. These programs predict and rank the potential off-target sites on the basis of the degree of similarity to the on-target site, position, and the type of mismatch within the protospacer sequence. Computationally predicted off-target sites are analyzed with mismatch detection assays (CEL 1 mutation detection assay and T7 endonuclease 1 assay) or Sanger sequencing. However, DSB generated outside the predicted sites are undetectable by these methods and examining a large number of loci for off-target mutations by mismatch detection assays is neither a practical nor a cost-effective strategy. Alternate sensitive methods have been developed that use different strategies for detecting bona fide off-target mutations in an unbiased manner but none of the methods can detect off-target effects comprehensively. dCas9 ChIP assays use a catalytically dead version of Cas9 to determine the genome-wide binding profile of dCas9 with a specific gRNA (Figure 6(A)). dCas9 ChIP followed by deep sequencing identifies the on-target site and other genome-wide Cas9 binding sites [82]. However, Cas9 binding is more promiscuous than its cleavage activity: most of the off-target DNA binding sites recognized by dCas9 are not cleaved at all by Cas9 in cells [83]. Genome-wide screening of DSB by unbiased identification of DSBs enabled by sequencing (GUIDE-seq) [84] (Figure 6(B)) and integration-deficient lentiviral vector (IDLV) [85] (Figure 6(C)) are sensitive methods for the detection of genome-wide off-target cleavages by Cas9. Techniques of detection by GUIDE-seq and IDLV rely on the NHEJ-mediated integration of small duplex oligonucleotides and lentiviral vectors, respectively, at cleavage sites. Clustered sites of integration in GUIDE-seq and IDLV are recovered by linear amplification-mediated STAT-PCR and LAM-PCR, respectively, and then mapped using high-throughput sequencing. Comparatively, GUIDE-seq is highly sensitive as compared to IDLV capture and can detect off-target sites with indel rate of 0.1% or lower in a population of cells [84].

Genome-wide off-target DSBs can also be captured in fixed, permeable cells by an unbiased detection method called BLESS (direct in situ breaks labelling, enrichment on streptavidin, and next-generation sequencing) [86, 87] (Figure 6(D)). BLESS directly labels genome-wide unrepaired DSBs by ligating biotinylated linkers which are then enriched and subjected to amplification and sequencing. Because this method can only capture DSBs at a specific moment, many bona fide off-target DSBs can be missed resulting in poor sensitivity [58, 88]. Another approach, Digenome-seq (digested genome sequencing), has been developed that relies on whole genome sequencing (WGS) of cell-free genomic DNA digested* in vitro* using Cas9-sgRNA nuclease complex [89]. Digenome-seq is a reproducible and sensitive method for the detection of off-target effects in an unbiased manner. This method can detect off-target sites at which indels are induced with a frequency of 0.1% or less. Another method of detecting genome-wide DSBs generated by RGNs is high-throughput, genome-wide translocation sequencing (HTGTS) [90] (Figure 6(E)). This method is based on the ability of the DSB generated by RGN to translocate to a fixed “bait” DSB generated by yeast I-SceI and to induce the end joining between the known “bait” sequence and unknown “prey” sequence in mammalian cells. Translocation events are subjected to LAM-PCR by using a biotinylated primer against a known “bait” sequence followed by streptavidin-based enrichment and subsequent high-throughput sequencing [90, 91]. This method is sensitive, robust, reproducible, and cost-effective and allows in-depth studies of mechanisms by which prey DSBs translocate to bait DSBs. Genome-wide off-target mutations with RGNs can also be assessed by whole genome sequencing (WGS) [92]. Off-target sites in edited cells can be determined by directly comparing the whole genome of nuclease-treated clone with the parental line via WGS [92, 93]. Although this method is useful for the analysis of single cell clone and F1 genome edited animals, it lacks the feasibility to analyze a large number of nuclease-treated clones which means that most off-target events that occur with low frequency in a population of cells are missed [94]. Furthermore, this method is economically expensive, technically difficult, and tiresome for assessing changes in the genome [95].

## 6. Delivery of CRISPR/Cas9 Components for Genome Editing

**Figure 7:**
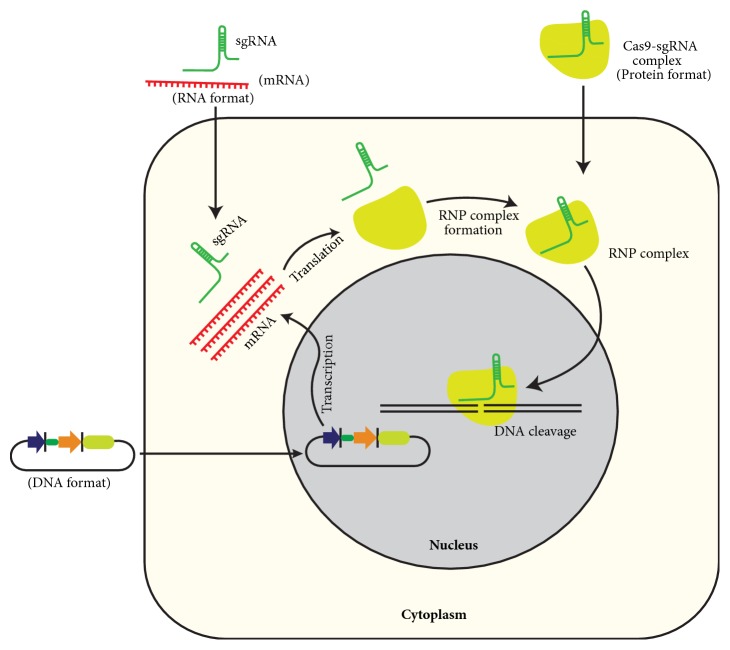
**Different formats for gene editing using CRISPR/Cas9 in cultured cells:** CRISPR/Cas9 components are delivered in DNA, RNA, or RNP formats. Delivery as RGN-ribonucleoproteins (RNPs) improves efficiency and specificity and sidesteps other limitations associated with the use of plasmids.

CRISPR/Cas9 cargo is delivered in different formats for genome editing in cells: DNA expression vectors encoding Cas9 and sgRNA from the same or separate vectors; in vitro transcribed Cas9 mRNA and sgRNA or as RNP complex of Cas9 recombinant protein and sgRNA (Figure 7). However, delivery of recombinant Cas9 protein and sgRNA as RNP complex offers considerable advantages over plasmid-based and Cas9 mRNA-sgRNA based delivery (Table 2).

Delivery of CRISPR/ Cas9 cargo is the biggest obstacle in achieving efficacious genome editing, especially* in vivo* for therapeutic purpose [96]. In cultures, CRISPR cargo is commonly delivered by lipofection, electroporation, nucleofection, and microinjection. Viral vectors such as lentivirus, adenovirus, and adeno-associated virus (AAV) are broadly used as delivery vehicles of CRISPR cargo for efficient genome editing* in vivo *[97]. However, delivery by lentivirus and adenovirus can elicit potential immune responses. Lentivirus is also associated with the risk of integration into the host genome [98]. AAV, which is favoured for gene delivery* in vivo*, is associated with minor immune responses and little pathogenesis. However, the cargo capacity of AAV is limited (~4.5 kb only) [99], and the most commonly used form of Cas9 from* Streptococcus pyogenes* and sgRNA with efficient promoters is difficult to package in typical AAV construct. To overcome this limitation, Cas9 and sgRNAs with their regulatory elements are packaged into separate vectors that increase the overall packaging capacity. In another strategy, Cas9 is splitted and packaged into parallel AAV particles; each Cas9 part is fused to a split intein which fuses the Cas9 parts together upon coexpression [100]. However, codelivery of separate vectors can reduce the editing potential.

Currently, a form of Cas9 from* Staphylococcus aureus* is used for* in vivo* genome editing with an efficiency similar to that of spCas9 while being more than 1 kb shorter, so that Cas9 and its sgRNA expression cassette can be packed into a single AAV genome editing vector [87]. Two smaller CRISPR proteins CasX and CasY have been discovered in bacteria which can further facilitate the delivery of CRISPR cargo by AAVs for genome editing [101]. Recently, Lee at al. demonstrated the efficient and localized delivery of Cas9 and Cf1 ribonucleoproteins by using gold nanoparticles conjugated to single stranded DNA for correcting genetic diseases and neurological disorders* in vivo *without any discernible impact [102].

## 7. CRISPR/Cas9 Approach for Transcriptional Activation and Suppression of Genes

**Figure 8:**
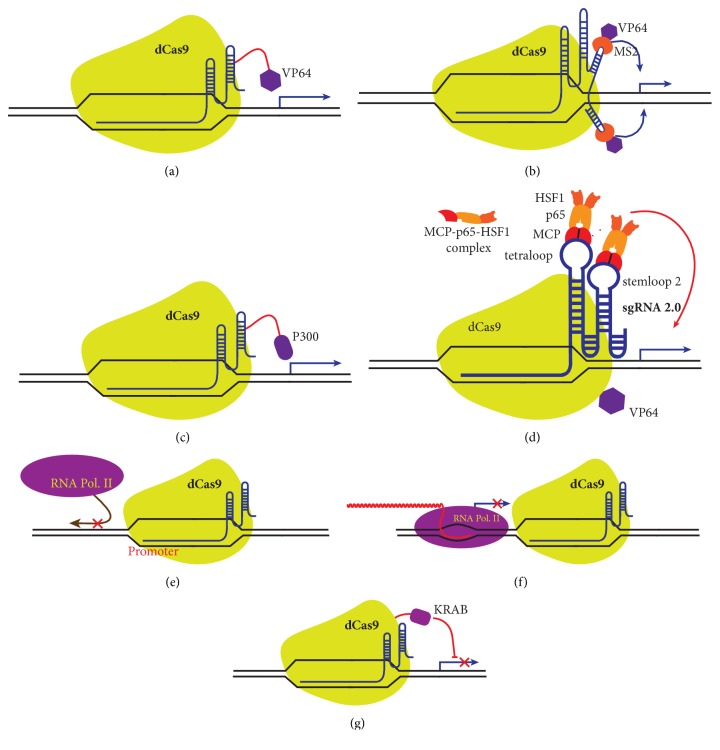
**Overview of Cas9 approach for transcriptional activation and repression of gene/s. (a) **RNA guided transcription activation can be achieved by fusing the dCas9 with VP64.** (b) **Transcription activation by chimeric sgRNA, capable of recruiting activation domains (MS2-VP64).** (c)** Transcriptional activation by the dCas9-p300 system.** (d) **SAM based transcriptional activation of endogenous genes. The SAM complex consists of dCas9-VP64 complex, sgRNA with two MS2 RNA aptamers, and MS2 RNA-binding protein (MCP) fused to activators p65 and HSF1.** (e) **Fusion of dcas9 with KRAB repressor can be used to achieve transcriptional repression.** (f) **Cas9 acts as a repressor by sterically blocking the transcription machinery.** (g)** Cas9 acts as a repressor by blocking transcription elongation.

Cas9 nuclease protein has the remarkable feature of being able to bind itself to gRNA directed target sites independent of its ability to cleave DNA. Mutations in the nuclease domains of Cas9 (RuvC (D10A) and HNH (H840A) result in a nuclease-null deactivated variant of Cas9(dcas9) that can be exploited to regulate transcription of the desired gene [103, 104]. dCas9 can be converted into sgRNA guided transcription activator (dcas9-activator) by tethering VP64, an engineered tetramer of the herpes simplex VP16 transcriptional activator domain, to the C-terminus of dCas9 [105, 106]. VP64 in dCas9-VP64 complex recruits multiple components of a preinitiation complex which when combined with gRNA targeting sequences near the promoter robustly activates gene expression [107] (Figure 8(a)). In another approach, VP64-MS2 fusion protein is recruited to the stem loops of sgRNA which selectively bind the dimerized MS2 bacteriophage coat proteins 1. Expression of chimeric sgRNA together with dCas9 and the MS2-VP64 fusion protein is more potent in upregulating endogenous gene expression as compared to sgRNA with dCas9-VP64 [105] (Figure 8(b)). In the dCas9-P300 activator system, which consists of a dCas9 protein fused to the catalytic core of histone acetyltransferase p300, catalyses the histone acetylation for robust gene activation from promoters and both proximal and distal locations relative to the transcription start site through chromatin remodelling [108] (Figure 8(c)). Konermann et al. have developed an advanced dCas9-SAM activation system by engineering an sgRNA2 containing two MS2 RNA aptamers at the tetraloop and stem-loop 2, capable of binding with dimerized RNA-binding protein MCP (MS2 coat proteins). Additional modifications of MCP by fusing the second and third activation domain, P65 and human heat-shock factor 1 (HSF1), form MCP–p65–HSF1 protein complex for robust transcriptional activation of coding as well as long intergenic noncoding RNA (lincRNA) through synergy. Coexpression of sgRNA2.0, NLS–dCas9–VP64, and MCP–p65–HSF1 has produced the most effective transcription activation system. It is designated as the synergistic activation mediator (SAM) (Figure 8(d)) [109].

dCas9 guided by gene-specific sgRNA can also be used to repress target genes [110, 111] by sterically blocking the transcription machinery. Programmed dCas9 binds the target site at promoter or coding region of the gene to act as a repressor by aborting transcription initiation (Figure 8(e)) and elongation (Figure 8(f)). This dCas9 directed silencing of transcriptional activity, termed CRISPR interference (CRISPRi), can be exploited to specifically silence target genes in both prokaryotic and eukaryotic cells. In bacterial cells, dCas9 alone can efficiently silence the transcription of the target gene, usually in the range of 1000-fold [110]. Effects of repression can be reversed by using inducible promoters to control the expression of dCas9.

In mammalian cells, the combined activity of dcas9 and the transcription repressor, Krüppel associated box (KRAB), in a dCas9-KRAB fusion protein complex allows an efficient, heterochromatin-mediated transcription repression of a target gene by using the appropriate gRNA [106, 112] (Figure 8(g)). This efficient CRISPRi activity is achieved by targeting the dCas9-KRAB fusion protein to a window of DNA from -50 to +300 bp relative to the transcription start site (TSS). So far the knockdown of mRNA by RNAi has been the dominating genetic tool for defining the function of the gene and has been used in several applications. RNAi is limited to depletion of cytoplasmic mRNA targets whereas CRISPRi can target elements across the entire genome, including enhancer RNAs, upstream antisense RNAs, and lncRNAs as well as other intergenic RNAs. It also offers less off-target effects in comparison with RNAi.

## 8. Applications and Recent Advances in Genome Editing Using the CRISPR/Cas 9 System

In the recent past, researchers have explored CRISPR/Cas9 gene editing technology to combat HIV infection. Latently infected CD4^+^ T cell lines, primary CD4^+^ T cells, and induced human pluripotent stem cells (iPSCs) were incorporated with Cas9 and targeting gRNAs which prevents against the new infection by HIV-1 [113–115]. CCR5 and CXCR4 coreceptor genes have also been edited to render cells refractory to HIV-1 infection [116, 117]. However, the emergence of replication-competent viruses that are resistant to Cas9/sgRNA was also observed [118, 119]. This technology was also employed to excise the integrated HIV-genome from the infected cells of preclinical animal models, including “humanized” mouse, using saCas9 and multiplex sgRNA [120], a step that is important towards fighting an HIV infection.

This system has successfully corrected genetic disease-causing mutations in human cell lines for cystic fibrosis [121, 122] and *β*-thalassemia [123]. It was also investigated that mice with mutations in the* Crygc* gene and dystrophin gene* (dmd)* that causes cataracts and Duchenne muscular dystrophy (DMD), respectively, could be rescued by coinjection of Cas9 mRNA and sgRNA targeting the mutant allele into zygotes [124, 125]. Delivery of CRISPR/Cas9 components through the hydrodynamic injection or adeno-associated virus-9 have been applied to correct mutation of fumarylacetoacetate hydrolase* (FAH)* or dystrophin gene* (dmd) *in mouse models of hereditary tyrosinemia type I (HTI) or Duchenne muscular dystrophy through homologous recombination or exon skipping therapy [126–128].

Engineered CART cells deleted with programmed cell death-1 (PD-1) or cytotoxic T lymphocyte-associated protein 4 (CTLA4) receptor were delivered into patients for enhancing the antitumour activity of CAR T [129]. Allogeneic T cells are used for the generation of universal CAR T cells with disrupted TCR, b-2 microglobulin, and PD-1 receptor that led to reduced alloreactivity and enhanced the anti-tumour activity of CAR T cells in mouse models [130]. This technology is also applied for CD19-specific CAR coding sequence into the T cell receptor *α* constant (TRAC) locus to create CAR T cells for cancer therapy [131]. The first clinical trial of CART cells has started in China for the treatment of lung cancer (CYRANOSKI D).

CRISPR editing approaches have been applied for the correction of common *β*-hemoglobinopathies, including sickle cell disease (SCD) [132, 133] and *β*-thalassemia disorders [134] as well as an immunodeficient disorder (chronic granulomatous disease) caused by defect in NADPH oxidase 2 (NOX2) protein [135].

CRISPR/Cas9 mediated gene editing can allow one-step generation of double gene mutant mice carrying biallelic mutations and disruption of multiple genes in mouse embryonic stem cells simultaneously as well as generation of mice carrying endogenous reporters [115, 136]. This technology can also be used to achieve efficient genome editing into specific organs and tissues postnatally to study the function of essential genes that cause embryonic lethality [137] and can be successfully experimented to disrupt genes in rats [138, 139], pigs [140], rabbits [141], dogs [142], and monkeys [143–145].

This technology has been used to edit the mutations in human embryos causing *β*-thalassemia [146] and hypertrophic-cardiomyopathy [147]. CRISPR/Cas9 technology is also used to introduce extensive DNA modifications into pigs for xeno-generating transplantable human tissues and organs by eradicating all porcine endogenous retroviruses (PERVs) and other genes for proteins that provoke cross-species immune rejection [148, 149].

A CRISPR/Cas9 mediated gene drive can be used to eliminate malaria by creating genetically engineered mosquitos [150, 151]. However, implementation of gene drive to wipe out invasive species or for altering the wild population has bred much controversy particularly with regard to environmental consequences of species extirpation. This technology is also ideally suited for genome-wide screens with sgRNA libraries for gene knockouts [152–154]. A genome-wide knockout study in human cell lines can completely disrupt target genes, thus avoiding weak signals that can occur when transcript abundance is partially decreased by siRNA [152, 153]. Pooled CRISPR screens the genes that confers cancer drug resistance [152, 153, 155], cancer cell proliferation [156, 157], development of metastasis [158], cancer-specific synthetic lethal targets [159], essentiality of genes in cancer immunotherapy [160], bacterial toxin resistance [161], and genes that influence the immune response [162].

In addition, CRISPR activation (CRISPRa) and CRISPR interference (CRISPRi) for the gain- or loss-of-function studies have also been adapted as a premier tool for high-throughput screening that generates robust and complementary data for mapping complex biochemical and cell signalling pathways [106, 109, 163].

## 9. Concluding Remarks

The CRISPR/Cas9 system has developed rapidly since its introduction in 2013 and is gaining momentum in the correction of disease mutations, combating viral diseases, dissecting gene function, cancer research, genome engineering of cells, drug discovery, and disease modelling. Despite being a revolutionary tool and despite causing major upheavals in the biological sciences, the technology has encountered challenges such as off-target effects, delivery, and the high frequency of indel formation over the desired HDR editing.

However, landscapes around Cas9 for improving specificity and applicability are rapidly evolving through Cas9 engineering and delivery to achieve the precise and efficient genome editing. Also, the characterization of small RGN in a biological diversity that is highly specific and easier to deliver needs to be explored. It is also required that an effective technique for bias DSB repair towards HDR for precise genome editing be developed as the strategies for achieving efficacious HRD repair are still encountering limitations. Despite these hurdles, this technology has been making headlines and it can be safely predicted that in future it will revolutionize biology and will change the world for the better.

## Figures and Tables

**Table 1:** Computational tools available for designing guide RNAs.

Name	Homepage	Available RGN	Off-target scoring	Predicts gRNA activity	Off-target hits	References
Cas-OFFinder	http://www.rgenome.net/cas-offinder/	7	no	no	yes	[44]

Benchling	https://benchling.com/	1	yes	yes	yes	

Deskgen	https://www.deskgen.com/landing/	1	yes	yes	yes	

CRISPR Design	http://crispr.mit.edu/	1	yes	no	yes	[45]

E-CRISPR	http://www.e-crisp.org/E-CRISP/	1	yes	yes	yes	[46]

CHOPCHOP	http://chopchop.cbu.uib.no/	2	no	yes	yes	[47, 48]

ZiFiT	http://zifit.partners.org/ZiFiT/CSquare9Nuclease.aspx	1	yes	no	yes	[49]

sgRNA Designer	https://portals.broadinstitute.org/gpp/public/analysis-tools/sgrna-design	2	no	yes	yes	[50, 51]

CRISPRseek	http://bioconductor.org/packages/release/bioc/html/CRISPRseek.html	1	yes	yes	no	[52]

CRISPR-Era	http://crispr-era.stanford.edu/	1	yes	yes	no	[53]

*∗*Supplementary file: workflow to design *in silico* gRNAs.

**Table 2:** Delivery of CRISPR/Cas9 components in different formats.

	Plasmid DNA	Cas9 mRNA + sgRNA	Cas9 Protein + sgRNA
Efficiency	++++	++	++
Specificity	++	++++	++++
Cell mortality	++++	+	+
Off-targets	++++	+	+
Stress to cells	++++	+	+
Onset of action	+	++	++++
Half life	++++	++	+
Risks of random integration	++++	#	#

#, no risk of integration.

## Supplementary Materials

Supplementary Materials In silico designing of custom gRNAs 1. Open the ensemble homepage (https://www.ensembl.org/) 2. Search and open the candidate gene of a species. 3. Open the first transcript and click exons on the upper left side of a page. 4. Scroll down and note the ID of different exons of a gene. 5. Repeat the process for other protein coding transcripts. 6. Fetch target exon/exons close to 5' end that is/are common to all transcripts. 7. Copy the exon sequence and paste in CRISPR DESIGN tool (http://crispr.mit.edu/) 8. Choose the appropriate gRNAs and add 5' overhang “CACC” for sense CRISPR strand and “AAAC” to the antisense strand for cloning in pSpCas9(BB)-2A-Puro V2.0 (Addgene PX459) and pSpCas9(BB)-2A-GFP (Addgene PX-458). For the gRNA that do not start with guanidine, add G to the 5' end of gRNA to improve U6 mediated transcription. 9. Analyze the targeted domains that define distinct function using InterPro (https://www.ebi.ac.uk/interpro/) and Motif (http://www.genome.jp/tools/motif/). 10. Use other gRNA design tools to evaluate the effectiveness of gRNAs (Table 1).
