# Supplementary material for: CRISPR/Cas9 System: A Bacterial Tailor for Genomic Engineering
Source: Genet Res Int. 2018 Sep 18;2018:3797214. doi: 10.1155/2018/3797214 (PMC6167567; doi:10.1155/2018/3797214)
Supplement: Supplementary Materials — In silico designing of custom gRNAs 1. Open the ensemble homepage (https://www.ensembl.org/) 2. Search and open the candidate gene of a species. 3. Open the first transcript and click exons on the upper left side of a page. 4. Scroll down and note the ID of different exons of a gene. 5. Repeat the process for other protein coding transcripts. 6. Fetch target exon/exons close to 5' end that is/are common to all transcripts. 7. Copy the exon sequence and paste in CRISPR DESIGN tool (http://crispr.mit.edu/) 8. Choose the appropriate gRNAs and add 5' overhang “CACC” for sense CRISPR strand and “AAAC” to the antisense strand for cloning in pSpCas9(BB)-2A-Puro V2.0 (Addgene PX459) and pSpCas9(BB)-2A-GFP (Addgene PX-458). For the gRNA that do not start with guanidine, add G to the 5' end of gRNA to improve U6 mediated transcription. 9. Analyze the targeted domains that define distinct function using InterPro (https://www.ebi.ac.uk/interpro/) and Motif (http://www.genome.jp/tools/motif/). 10. Use other gRNA design tools to evaluate the effectiveness of gRNAs (Table 1). [file 3797214.f1.docx]

***In silico* designing of custom gRNAs**

1. Open the ensemble homepage (<https://www.ensembl.org/>)
2. Search and open the candidate gene of a species.
3. Open the first transcript and click exons on the upper left side of a page.
4. Scroll down and note the ID of different exons of a gene.
5. Repeat the process for other protein coding transcripts.
6. Fetch target exon/exons close to 5' end that is/are common to all transcripts.
7. Copy the exon sequence and paste in CRISPR DESIGN tool (<http://crispr.mit.edu/>)
8. Choose the appropriate gRNAs and add 5' overhang “CACC” for sense CRISPR strand and “AAAC” to the antisense strand for cloning in pSpCas9(BB)-2A-Puro V2.0 (Addgene PX459) and pSpCas9(BB)-2A-GFP (Addgene PX-458). For the gRNA that do not start with guanidine, add G to the 5' end of gRNA to improve U6 mediated transcription.
9. Analyse the targeted domains that defines distinct function using InterPro (<https://www.ebi.ac.uk/interpro/>) and Motif (<http://www.genome.jp/tools/motif/>).
10. Use other gRNA design tools to evaluate the effectiveness of gRNAs(Table I).
